# Current status and challenges of breast cancer prevention~DNA methylation would lead to groundbreaking progress in breast cancer prevention~

**DOI:** 10.1186/s41021-023-00287-0

**Published:** 2023-12-12

**Authors:** Takahiro Tsukioki, Seema A. Khan, Tadahiko Shien

**Affiliations:** 1 Department of General Thoracic Surgery and Breast and Endocrine Surgery, Okayama University Graduate School of Medicine, Dentistry, and Pharmaceutical Sciences, 2-5-1 Shikata-cho, Kita-ku, Okayama, 7008558 Japan; 2 Department of Surgery, Feinberg School of Medicine of Northwestern University, 303 East Superior Street, Chicago, IL 60614 USA

**Keywords:** Breast cancer, Prevention, Risk reduction mastectomy, Chemoprevention, Methylation

## Abstract

The number of breast cancer patients is increasing worldwide. Furthermore, breast cancer often develops in young people, even those only in their 30s, who play a central role in their families and society. Results from many cohort studies suggest that dietary factors, alcohol consumption, lack of physical activity, obesity, nulliparity, breastfeeding, oral contraceptive use, fertility treatment and hormone replacement therapy are risk factors for breast cancer. However, the effects of lifestyle habits on the human body are complexly intertwined with various factors, and the effects vary from person to person depending on their constitution, etc., so there is no basis for this. Therefore, primary prevention of breast cancer is still not being implemented appropriately and efficiently. Furthermore, advances in genomic technology make it possible to assess the risk of developing breast cancer in some individuals. As a result, the establishment of breast cancer prevention methods has become a health priority for high-risk individuals.

Drugs such as tamoxifen and raloxifene are known to prevent the development of breast cancer, based on the results of multiple randomized controlled trials, but there are concerns regarding the side effects of these powerful agents. In addition, several clinical studies have shown that prophylactic mastectomy for women who have BRCA mutations or who are identified as being at high risk reduces the incidence of breast cancer development. However, many issues, such as changes in long-term quality of life after preventive surgery, the optimal timing of surgery and the identification of women who are at high risk but will not develop breast cancer, remain uncertain. In other words, although many researchers have focused on chemoprevention and surgical prevention and clear preventive effects of these strategies have been confirmed, it cannot be said that they are widely accepted. Therefore, the current evidence for chemoprevention and surgical prevention, as well as highlights of several interesting lines of research currently underway, are summarized in this article.

## Background

Breast cancer (BC) incidence has been rising annually and is now the most prevalent cancer in women in many countries, as well as the most common cancer in young women. Heer and colleagues reported BC mortality and long-term trends in 41 countries. Their report showed that approximately 2.05 million people were diagnosed with BC in 2018, accounting for 24% of cancers in women and 15% of deaths [1]. Therefore, establishing preventive strategies against BC development is an urgent issue.

The most important thing for BC prevention is assessing a risk of developing BC, and then, we need to recommend some prevention methods according to their risk level, because chemoprevention and surgical prevention are associated with side effects. In other words, prevention strategies without side effects, such as lifestyle changes, weight loss, and physical activity, are applicable to all women, and chemoprevention are considered in women at moderate risk, and surgical prevention are suggested at very high risk of BC development.

## Assessment of developing BC risk

Due to advances in research in the field of genetics have revealed that certain genes are strongly associated with the development of cancer. Furthermore, advances and prevalence of genetic testing and analysis of accumulated data indicate that approximately 10% of BC patients carry germline mutations. We already know that germline protein truncation variants (PTV) and rare missense variants (MSV) in nine genes, i.e., AMT, BRCA1, BRCA2, CHEK2, PALB2, BARD1, RAD51C, RAD51D, and TP53, are reportedly strongly associated with BC development [2]. In addition, a database study revealed associations between each of these gene mutations and BC subtypes. RAD51C, RAD51D, and BRCA1 are associated with the development of triple-negative BC, AMT with luminal BC, and TP53 with ERBB2 (HER2)-positive BC [3]. From the aspect of secondary prevention, assessing the genetic risk of BC and recommending genetic testing offer major benefits. This is because providing early medical intervention for those with mutations is highly significant in terms of improving the survival prognosis. We provide genetic counseling for BC patients considered to be at high genetic risk, and we recommend genetic testing if there is a possibility of genetic mutations. Women with these genetic mutations are at increased risk of developing BC and require surveillance such as mammography and magnetic resonance imaging (MRI) screening. In addition, it is important to consider chemoprevention and risk-reducing surgery [4, 5].

On the other hand, the fact that genomic gene related BC accounts for only about 10% of cases suggests that environmental factors (epigenetic change) are strongly involved in the development of BC. For women who are negative or low risk for hereditary BC, we need to some tools or risk models for assessing their risk of developing BC. In the USA and Europe, BC risk predictive models, such as the Gail model and other models that have improved detection rates by incorporating whole genome analysis data are used to evaluate the risk of developing BC over a period of 5 years [6–10]. It is important to implement preventive strategies according to those individual risk levels. In the other words, to assess your risk of developing breast cancer, you should first thoroughly investigate your family history of breast and ovarian cancer, and if you suspect that you have hereditary breast cancer, you should actively consider genetic test. If you determine that familial breast cancer is unlikely, use existing risk models to assess your breast cancer risk, focus on primary prevention, and work with your healthcare provider to develop appropriate prevention strategies based on your risk level.

We review existing evidence for surgical and chemoprevention and new BC prevention research.

## Prevention strategies applicable to all women

BC is the most common cancer among women in many countries. Even women who are judged to have a low risk of developing BC should receive annual medical examinations and strive to detect BC at an early stage, and it is important to practice primary prevention such as lifestyle, weight loss, physical activity and so on.

As for the primary prevention of BC, the relationship between BC and food/nutrition has been studied and several reports have been published to date, mainly in Western countries. Based on these lines of evidence, the World Cancer Research Fund (WCRF) and the American Institute for Cancer research (AICR) have evaluated and reported causal relationships with the development of BC [http://www.dietandcancerreport.org]. Although BC risk factors might differ between the pre- and post-menopausal states, alcohol, obesity, and height are widely regarded as risk factors. On the other hand, physical activity, breastfeeding history, and consumption of non-starchy vegetables and foods, high in carotenoids and calcium, are considered to be risk-reducing factors, but the associations with many other factors are as yet unclear [11]. Primary prevention is considered to be very beneficial, but the efficacy of improving dietary and exercise habits, and the preventive effects of such improvements, remain uncertain.

## Prevention for moderate risk women

Women who are determined to be at low risk of hereditary BC but at moderate or higher risk of developing BC should continue medical examinations and consider more aggressive preventive measures such as chemoprevention.

Since the efficacy of chemoprevention in women at moderate and high risk of developing BC was reported in 1998, a number of placebo-controlled randomized trials have been reported. The results of these trials revealed that chemoprevention decreased BC development [12].

## Chemoprevention

### Selective estrogen receptor modulators (SERM)

#### Tamoxifen (TAM)

Four randomized controlled trials have compared the BC preventive effect of TAM with that of a placebo: NSABP P-1, IBIS-I, Royal Marsden Hospital Tamoxifen Prevention Trial, and the Italian Randomized Tamoxifen Prevention Trial. According to meta-analyses of these trials, TAM produced a 30% reduction in invasive ductal carcinoma (IDC) development [13–16].

#### Raloxifene

Three randomized controlled trials (MORE, CORE, RUTH) have investigated the efficacy of raloxifene in preventing BC development, as compared with a placebo. Meta-analyses of the data obtained showed a 56% reduction in IDC [17–19].

In the STAR trial, which directly compared the effects of TAM and raloxifene on breast cancer prevention, the RR was 1.24 (95% CI 1.1–1.5), being significantly lower in the TAM group [20]. In summary, SERM significantly reduced the development of IDC (mainly that of hormone receptor-positive BC) in high-risk women who have a history of lobular carcinoma in situ (LCIS) and who were judged to be at high risk according to the Gail model. The serious side effects include endometrial cancer and thrombosis, and the effectiveness of low-dose chemoprevention to overcome these side effects has also been reported. The results of ongoing clinical research on treatments and therapeutic agents with fewer side effects and higher preventive efficacy are awaited. We are eager to develop treatments and preventive agents that have minimal side effects and are highly effective.

### Aromatase inhibitors (AI)

#### Exemestane (EXE)

Mammary Prevention 3 trial, a randomized controlled study of exemestane for preventing the development of IDC in postmenopausal women at high risk of developing BC, found exemestane to have a superior protective effect [HR 0.35 (95% CI 0.18–0.7)] [21].

#### Anastrozol (ANA)

Similarly, International Breast Cancer Intervention Study II, a placebo-controlled randomized controlled trial targeting postmenopausal women at high risk of breast cancer, found a significant reduction in the risk of developing invasive breast cancer in the ANA group. [HR 0.58 (95%CI 0.39–0.66)] [22].

As I summarize, several drugs have been reported to be effective in preventing the development of BC (Table 1). However, fear of side effects and lack of understanding of BC, only 4.1% of women at high risk of developing BC are willing to take chemo preventive drugs. In other words, due to the side effects of chemoprevention (hormone therapy) such as menopausal disorders, joint stiffness, thrombosis and endometrial cancer and because we do not know the most appropriate time to start chemoprevention and how risk changes after its completion, this strategy is not widely accepted. Future tasks include mitigating drug side effects, assessing the appropriate timing to initiate chemoprevention, and considering the duration of chemoprevention.

**Table 1:** Randomized controlled trials of chemoprevention

Drug	First author	Year	Prevention drug	Study design	Patient characteristics	Total patient number	Number of patients given drug	Number of patients given placebo	Follow up(months)	Development of BC	Development of IDC	Development of DCIS
TAM	Bernard Fisher	2005	Placebo vs TAM 20mg(5years)	Randomized controlled trial	>60 years or between 35 and 59 years with high risk of BC, or a history of LCIS or atypical hyperplasia	13388	6681	6707	74		RR; 0.57 (95% CI: 0.46-0.7)	RR 0.63 (95% CI; 0.45-0.89)
	Jack Cuzick	2014	Placebo vs TAM 20mg(5years)	Randomized controlled trial	35–70 years with high risk of BC	7154	3579	3575	192	HR; 0.71 (95% CI: 0.6-0.83)		
	Powles TJ	2007	Placebo vs TAM 20mg(8years)	Randomized controlled trial	30-70 years with high risk of BC	2471	1238	1233	158	HR 0.84 (95% CI; 0.64-1.1)	HR 0.78 (95% CI; 0.58-1.04)	-
	Umberto Veronesi	2007	Placebo vs TAM 20mg(5years)	Randomized controlled trial	35–70 years with high risk of BC	5408	2708	2700	109	HR; 0.84 (95% CI: 0.6-1.17)		
Raloxifene	J A Cauley	2001	Placebo vs Raloxifene 60mg vs 120mg	Randomized controlled trial	postmenopausal women with osteoporosis and no history of BC	7705	2557(60mg), 2572(120mg)	2576	40		RR 0.28 (95% CI; 0.17-0.46)	
	Silvana Mrtino	2004	Placebo vs Raloxifene 60mg	Randomized controlled trial	postmenopausal women with osteoporosis and no history of BC	4011	2725	1286	96	HR 0.42 (95% CI; 0.29-0.6)	HR 0.34 (95% CI; 0.22-0.5)	
	Elizabeth Barrett-Connor	2006	Placebo vs Raloxifene 60mg	Randomized controlled trial	postmenopausal women with CHD or multiple risk factors for CHD	10101	5044	5057	66	HR 0.67 (95% CI; 0.47-0.89)	HR 0.56 (95% CI; 0.38-0.83)	HR 2.17 (95% CI; 0.75-6.24)
Exemestane	Paul E. Goss	2011	Placebo vs Exemestane	Randomized controlled trial	>60 years with high risk of BC, or a history of DCIS, LCIS, atypical ductal or lobular hyperplasia	4560	2285	2275	35	HR 0.47 (95% CI; 0.27-0.79)	HR 0.35 (95% CI; 0.18-0.7)	HR 0.65 (95% CI; 0.28-1.51)
Anastrozole	Jack Cuzick	2014	Placebo vs Anastrozole	Randomized controlled trial	40–70 years with high risk of BC	3851	1914	1937	60	HR 0.47 (95% CI; 0.32-0.68)	HR 0.5 (95% CI; 0.32-0.76)	HR 0.3 (95% CI; 0.12-0.74)

## Prevention for high risk women

Stronger prevention strategies should be considered for high risk breast cancer development women who have BC-associated gene mutations and who are assessed at high risk by BC risk models. We need to consider surgical prevention or chemoprevention [4, 5].

## Surgical prevention for BRCA mutation women

Several gene mutations have been shown to correlate with BC. BRCA1 and 2 are considered to confer a particularly high risk for BC, and the prevalence of the BRCA1 mutation in BC patients is reported to be about 1.2% and that of BRCA2 about 1.5%. Women with BRCA1/2 variants have higher risks of developing BC by age 70 years, 64.6% (95% CI 59.5–69.4) and 61.0% (95% CI 48.1–72.5), respectively [23]. Therefore, it is critical to offer effective prevention to these women. Surgical prevention includes contralateral risk-reducing mastectomy (CRRM) for BC patients and bilateral risk-reducing mastectomy (BRRM) for those who have never had BC.

### BRRM for individuals with a BRCA mutation

Two meta-analyses have demonstrated the efficacy of BRRM in preventing BC development in women with BRCA variants with no prior history of BC. Li reported that BRRM significantly reduced the relative risk (RR) of developing BC to 0.11 (95% CI 0.04–0.32) [24]. However, many of these cases also received risk-reducing bilateral salpingo-oophorectomy (RRSO), raising the possibility that RRSO itself suppresses BC development due to hormonal alterations. De Felice performed a meta-analysis that considered the effect of RRSO, and found that RR was 0.06 (95% CI 0.01–0.41) in the non-RRSO group and 0.11 (95% CI 0.01–0.86) in the RRSO group. BRRM was thus shown to significantly reduce the risk of BC development regardless of RRSO [25]. These meta-analysis also investigated survival. Each showed a tendency for improved survival, but the differences did not reach statistical significance (Table 2).

**Table 2:** Evidence supporting BRRM for women with BRCA mutations

First author	Year	Total patients in cohort study	BRCA1 patients	BRCA2 patients	Total number of BRRM patients	BRCA1 in BRRM	BRCA2 in BRRM	Median follow-up (year)	Development of BC (%)	RR (95% CI)	HR of all-cause mortality (95% CI)	HR of BC-specific mortality (95%CI)
Sarah L Ingham	2013	691	346	345	126	71	55	-	3%	0.11 (0.04-0.32)	0.25 (0.03-1.81)	0.29 (0.03-2.61)
B.A.M. Heemskerk-Gerritsen	2013	570	405	165	216	156	56	8.5	0	0.02 (0.00-0.33)	0.2 (0.02-1.68)	-
Hanne Meijers-Heijboer	2001	139	120	19	76	64	12	3	0	0.1 (0.01-1.42)	-	-
Susan M Domchek	2010	1619	1032	587	257	159	88	3.65	0	0.12 (0.01-1.87)	-	-
Skytte AB	2011	307	201	106	96	67	29	-	0.80%	0.49 (0.17-1.4)	-	-

### CRRM for individuals with a BRCA mutation

A meta-analysis concluded that CRRM performed in BC patients with a BRCA variant significantly reduced the risk of BC development with an RR of 0.07 (95% CI 0.04–0.15) [24]. In addition, five reports on survival rates have been published, and there was a meta-analysis in 2018. The latter showed significant mortality reduction. (HR 0.48, 95%CI 0.35–0.64) However, we must keep in mind that about 80% of the patients underwent RRSO. Thus, the possibility of RRSO impacting BC cannot be ruled out (Table 3).

**Table 3:** Evidence supporting CRRM for women with BRCA mutations

First author	Year	Total patients in cohort study	BRCA1 patients	BRCA2 patients	Total number of patients with BRRM	BRCA1 in BRRM	BRCA2 in BRRM	Median follow-up (year)	Development of BC (%)	RR (95% CI)	HR of all-cause mortality (95% CI)	HR of BC-specific mortality (95%CI)
TC Van Sprundel	2005	148	115	33	79	60	19	7.4	1.30%	0.04 (0.01-0.31)	0.35 (0.09-1.39)	3.80%
Evans	2013	718	357	361	105	-	-	7.2	4.90%	0.12 (0.02-0.88)	0.48 (0.19-1.14)	-
Metcalfe	2014	390	226	158	181	103	76	13	-	0.02 (0.00-0.18)	0.58 (0.34-0.97)	0.52 (0.29-0.93); 9.9%
Heemkerk-Gerritsen	2014	583	454	129	242	193	49	5.2	2%	0.14 (0.05-0.35)	0.49 (0.29-0.82)	-
Soenderstrup	2018	235	141	96	235	141	96	9.01	-	-	0.42 (0.21-0.84)	-

To summarize the evidence for surgical prevention in women with BRCA mutations, BRRM in non-BC patients has not been shown to improve survival, but is sufficiently effective for reducing BC development. Moreover, a meta-analysis of CRRM in BC patients demonstrated a reduced risk of developing contralateral BC and significant improvement of overall survival, although the possibility of RRSO impacting BC remains. For those with germline mutations, reducing the risk of BC is an important and actively accepted strategy both in terms of anxiety reduction and cost effectiveness. However, there are concerns regarding the lack of assessment of quality of life after surgical prevention, the optimal timing of surgery, and the identification of mutation-positive cases that do not develop BC [26].

### Chemoprevention for BRCA mutation women

There is no results and evidence from clinical trials that show chemoprevention efficacy in women with BRCA mutations and have not developed BC. NSABP-P1 trial, the largest randomized placebo-controlled trial of BC prevention, asked whether oral TAM for 5 years could prevent from developing breast cancer in women aged 35 and older who had never had BC. The result showed that 288 patients developed BC, and 8 patients had BRCA1 mutation [TAM: placebo = 5:3, RR: 1.67 (95% CI: 0.32–10.70)] and 11 had BRCA2 [TAM: placebo = 3:8, RR: 0.38 (95% CI: 0.06–1.56)] [27]. In short, there are only a few data showing a chemoprevention effect on the BC development in BRCA-mutated persons. Some clinical trial results are awaited.

### Surgical prevention for high risk women who are negative for gene-mutation

A lot of BC are apparently caused by epigenetic changes. According to the risk level, decisions are made regarding risk reduction surgery. Analysis using the SEER database indicates that the 25-year risk of contralateral BC development is about 10% in BC patients. In addition, contralateral BC risk does not differ by age at diagnosis or years since the initial BC diagnosis. This means that metachronous bilateral BC can be anticipated to occur with a probability of about 0.4% per year. There is no difference in the metachronous BC rate between DCIS (ductal carcinoma in situ) and IDC (intraductal carcinoma) cases [28]. BC patients must be monitored for and remain aware of the possible development of contralateral BC. Notably, Voralak reported that patients who developed contralateral BC within 5 years of their first BC had significantly poorer outcomes than those who developed these tumors 5 years or more after the first diagnosis of BC [29]. In addition, Schaapveld and colleagues reported that younger patients undergoing maintenance bilateral BC have poor outcomes, particularly those under the age of 40 [30, 31].

However, surgical prevention also has drawbacks. Kurian and colleagues reported a large retrospective cohort study and showed that the percentage of BC patients who chose prophylactic contralateral mastectomy increased from 2% in 1988 to 12% in 2011; the annual rate of increase was 14.3%. This tendency was particularly marked in cases under the age of 40 years, and gradually rose from 3 to 33% in his cohort. However, no prognostic improvements were derived from bilateral mastectomy versus treatment of only the affected breast [32, 33]. Further research to identify BC predictive markers and establish useful models is needed.

## Discussion: new directions in breast cancer prevention

As I summarize, it is clear that chemoprevention and prevention surgery reduce the risk of developing BC in women at moderate and high risk. However, it is not widely accepted due to concerns about side effects and inaccuracy of risk models. Research to reduce the side effects of chemoprevention and the construction of a risk model to more accurately determine the risk of developing BC due to epigenetic factors are challenges.

### Anticipated future chemoprevention

New strategies are currently being explored to reduce adverse side effects while preserving the beneficial anticancer properties of chemoprevention drugs. These studies include low-dose chemoprevention and gel-based topical drugs.

Lower doses of these drugs are expected to lead to fewer side effects, and the efficacy of low-dose TAM (1-5 mg) has been investigated in biomarker-based clinical trials and cohort studies. DeCensi and colleagues performed a randomized controlled trial to investigate the effect of low-dose TAM (5 mg) in preventing BC development in patients who received surgery for atypical ductal hyperplasia and intraepithelial lobular or ductal carcinoma. They reported that, clinically, low-dose TAM had beneficial preventive effects and fewer side effects than other regimens [12, 16, 34–37].

Transdermal drug delivery using gels has long been investigated as a potentially effective alternative to oral administration. Several studies have shown that topical drugs may be retained in local tissues for equal or longer durations than orally-administered agents [12, 38–42]. Oukseub reported the intramammary drug concentrations and distributions of topical and oral TAM. The topical TAM gel showed inferior drug concentrations in the breast but drug distributions were similar [43]. This suggests that topical agents might be a viable treatment option. Further research and clinical trial results are awaited, with the goals of overcoming side effects and identifying more acceptable forms of chemoprevention.

### Research on predictive markers for developing BC ~ Focusing on DNA methylation changes in mammary gland tissue~

Surgical prevention is highly invasive, so we need to consider risks and benefits. In other words, we need to be able to more accurately determine the risk of developing BC. To this end, it is important to identify epigenetic changes that effect developing BC. Epigenetic changes that affect gene expressions include genomic DNA methylation and demethylation, chromatin remodeling, histone methylation and acetylation, genomic imprinting, X chromosome inactivation, and noncoding RNA. Numerous investigations have obtained results suggesting that multiple forms of cell aging are caused by epigenetic changes, raising the possibility of a relationship between cell aging and cancer development [44–47].

Among these epigenetic mechanisms, an abundance of research has focused on DNA methylation. Many reports have shown that DNA methylation of CpG islands markedly impacts gene expressions. Epigenetic clocks calculated from the cumulative effects of DNA methylation might be useful in studies of developmental biology, cancer development and aging [48]. In other words, previous research reported that when DNA methylation occurs and accumulates in a specific region, it causes the inactivation of the gene, which is likely to cause developing BC. I summarize what we know from previous research (Table 4).

**Table 4:** Summary of methylation research using breast tissue

First author	Sample	DNA methylation kit	Finding
Erin W. Hofstatter	SQ, N	Illumina 450K	・DNA methylation Age of normal breast tissue was strongly correlated with chronological age
			・Compared to unaffected women, breast cancer patients exhibited significant age acceleration in their normal breast tissue
			・Smoking was positively correlated with epigenetic aging in normal breast tissue
James R. Castle	T, SQ, N	Illumina 27K and 450K	・DNA methylation age in Tumor was on average 7 years older than chronological
			・HER2(+) and HR(+) breast cancer demonstrated significant acceleration in DNA methylation ages, while there was no significant difference in triple-negative breast cancer
Kevin C. Johnson	T, SQ, N	Illumina 450K	・DNA methylation was not strongly associated with the other evaluated breast cancer risk factors instead of age
Chrstine B. Ambrosone	T, N	Illumina 450K	・Average methylation levels at loci within CGIs and CGI-shores were consistently higher in tumor than normal. On the other hand, these of loci outside of CGIs (ex: CGI-shelves and open sea) were lower in tumors.
			・Average methylation levels at loci within CGIs were higher in ER(+) tumors compared to ER(-) tumors
Min-Ae Song	N	Illumina 450K	・Methylation was correlated with expression of the corresponding gene and with DNA methyltransferase protein DNMT3A
			・Sites with increased methylation were predominantly in CpG islands and non-enhancers, and with decreased methylation were generally located in intergenic regions, non-CpG Islands, and enhancers
			・Expression of DNMT3A and KRR1 and DHRS12 were positively associated with age.
Andrew E. T	T, SQ, N	Illumina 450K	・Epigenetic field defects in breast cancer are widespread
			・Genomic distribution is highly non-random affecting binding sites of transcription factors specifying chromatin architecture and stem-cell differentiation pathways.
Bin Xiao	T, N	Illumina 450K and HiSeq 2000 RNA seq	・The correlation analysis of 122 methylation site–mRNA expression pairs revealed that 59 pairs were significantly correlated (42 were negatively and 17 were positively correlated)
			・ VIM, EPHX3, ACVR1, ANGPT1, TPM3, ALOX15, DIO1, KCNJ2, RSPH9, SOSTDC1, SYCP2, MACF1, TDRD5 and CELSR3 were significantly related to breast cancer prognosis
Xinhua Liu	T,SQ,N	Illumina 450K	・Lots of CpGs were hyper-methylated in breast cancer compared with adjacent normal tissues, which tend to be negatively correlated with gene expressions.
			・Eight CpGs located at RIIAD1, ENPP2, ESPN, and ETS1, were hyper-methylated in tumor

Several reports have compared the methylation of breast tissue in BC patients and normal breast tissue from women free of BC. In addition, previous research revealed the following. The age-related DNA methylation of unaffected women’s normal breast tissue is strongly correlated with chronological age, but BC patients exhibited significant age acceleration in their normal breast tissue. Moreover, DNA methylation-age in tumor was on average 7 years older than chronological [49, 50]. Smoking was positively correlated with epigenetic aging in normal breast tissue, but DNA methylation was not strongly associated with the other evaluated BC risk factors instead of age [51]. Average methylation levels at loci within CGIs and CGI-shores were consistently higher in BC tumor than normal breast tissue. On the other hand, these of loci outside of CGIs such as CGI-shelves and open sea were lower in tumors [52, 53]. CpG islands located at RIIAD1, ENPP2, ESPN, and ETS1, were hyper-methylated in BC tumor [52, 54, 55]. HER2(+) and ER(+) BC demonstrated significant acceleration in DNA methylation ages, while there was no significant difference in triple-negative BC [49–55]. However, these studies have some limitations and unclear points. First, most of the research having compared BC tissue with adjacent normal tissue, or being limited to comparisons with normal breast tissue from women without BC. In other words, several reports have shown that adjacent normal tissue is not actually normal because it is affected by the microenvironment of cancer [56]. Furthermore, it is also suspected that comparing methylation among different individuals may have limited value. Second, most of previous studies used public databases (TCGA or GEO) to identify DNA methylation involved in BC development. These methylations are information captured by Illumina’s Epic Methylation Array. In other words, it should be noted that this is not a genome-wide analysis. Furthermore, to translate these results into clinical practice, it would be desirable to be able to estimate the risk of developing BC using less invasive methods. In other words, the next step is to find factors in blood, saliva, urine, that accurately reflect the methylation changes in breast tissue associated with the development of BC, in order to create a risk model that can be easily used by all of women. Further research is expected to reduce or eliminate these limitations and enable the discovery and application of more accurate predictive markers, thereby establishing useful risk models.

If this happens, research into the effects of environmental factors on genes will accelerate, leading to breakthroughs that will rapidly advance preventive medicine for BC.

## Conclusion

BC is the most prevalent cancer in women worldwide, as well as the most common cancer in young women. Thus, preventing the development of BC is an important research theme. As a strategy for BC prevention, we have applied interventions such as changing dietary and exercise habits, surgery and chemoprevention. However, due to concerns about side effects and cosmetic issues, these strategies have not gained widespread acceptance. It is necessary to establish a preventive method that exerts high efficacy in preventing BC development while having minimal adverse effects. More accurate risk models and further research on acceptable surgical resection and chemoprevention methods are urgently needed.

## Data Availability

Not applicable.

## Abbreviations

BC: Breast cancer
PTV: Germline protein truncation variants
MSV: Rare missense variants
CRRM: Contralateral risk-reducing mastectomy
BRRM: Bilateral risk-reducing mastectomy
